# Structural Changes of Oil Palm Empty Fruit Bunch (OPEFB) after Fungal and Phosphoric Acid Pretreatment

**DOI:** 10.3390/molecules171214995

**Published:** 2012-12-17

**Authors:** Isroi, Mofoluwake M. Ishola, Ria Millati, Siti Syamsiah, Muhammad N. Cahyanto, Claes Niklasson, Mohammad J. Taherzadeh

**Affiliations:** 1 Department of Biotechnology, Graduate School of Gadjah Mada University, 55281 Yogyakarta, Indonesia; E-Mail: isroi93@gmail.com; 2 Department of Chemical and Biological Engineering, Chalmers University of Technology, 41296 Gothenburg, Sweden; E-Mail: claesn@chalmers.se; 3 School of Engineering, University of Borås, 50190 Borås, Sweden; E-Mail: mofoluwake.ishola@hb.se; 4 Department of Chemical and Polymer Engineering, Lagos State University, PMB 1012 Epe, Nigeria; 5 Department of Food and Agricultural Product Technology, Gadjah Mada University, 55281 Yogyakarta, Indonesia; E-Mails: ria_millati@ugm.ac.id (R.M.); mcahyanto@yahoo.com (M.N.C.); 6 Department of Chemical Engineering, Gadjah Mada University, 55281 Yogyakarta, Indonesia; E-Mail: ssyamsiah@chemeng.ugm.ac.id

**Keywords:** cellulose I_β_, crystallinity, digestibility, FTIR spectra, hydrogen bond, oil palm empty fruit bunch, phosphoric acid, *Pleurotus floridanus*, pretreatment

## Abstract

Oil palm empty fruit bunch (OPEFB) was pretreated using white-rot fungus *Pleurotus* *floridanus*, phosphoric acid or their combination, and the results were evaluated based on the biomass components, and its structural and morphological changes. The carbohydrate losses after fungal, phosphoric acid, and fungal followed by phosphoric acid pretreatments were 7.89%, 35.65%, and 33.77%, respectively. The pretreatments changed the hydrogen bonds of cellulose and linkages between lignin and carbohydrate, which is associated with crystallinity of cellulose of OPEFB. Lateral Order Index (LOI) of OPEFB with no pretreatment, with fungal, phosphoric acid, and fungal followed by phosphoric acid pretreatments were 2.77, 1.42, 0.67, and 0.60, respectively. Phosphoric acid pretreatment showed morphological changes of OPEFB, indicated by the damage of fibre structure into smaller particle size. The fungal-, phosphoric acid-, and fungal followed by phosphoric acid pretreatments have improved the digestibility of OPEFB’s cellulose by 4, 6.3, and 7.4 folds, respectively.

## 1. Introduction

Oil palm (*Elaeis guineensis*) is cultivated on more than 15 million ha land across the world, including 5.37 million ha in Indonesia [1]. Crude oil palm (CPO) is extracted from the fruits and the lignocellulosic residual remains as oil palm empty fruit bunch (OPEFB) at the mills. Indonesia is one of the largest oil palm producers in the world, where its production reached about 90 million metric tons of oil palm fruit in 2010 and as a result it accumulates about 20.7 million metric tons of OPEFB per year [1]. OPEFB has low commercial value and constitutes a disposal problem due to its large quantity. Conventionally, OPEFB is burned, disposed of in landfills, or composted to organic fertilizer. In order to prevent air pollution and other environmental problems, burning of OPEFB is not recommended. It is therefore of importance to optimally utilize OPEFB in order to solve these problems and at the same time utilize the resource for valuable products. OPEFB is mainly composed of 82.4% hollocellulose and 17.6% lignin [2]. Having high carbohydrate content, OPEFB has high potential as a source for lignocellulosic derived products, such as glucose, xylose, mannose [3], ethanol [4], biopulp [5], ruminant feed [6], and substrate for enzyme production [7].

For conversion of lignocelluloses into value-added products, pretreatment is an important step to enhance the digestibility of the materials. The enzymatic digestibility of lignocellulosic materials is limited by a number of factors such as lignin content and its composition, cellulose crystallinity, degree of polymerization, pore volume, acetyl groups bound to hemicellulose, surface area and biomass particle size [8,9,10]. This step is required to alter the structure of lignocellulosic biomass and break down the biomass recalcitrance to make cellulose more accessible to the hydrolytic enzymes. It is pointed out that delignification resulted in biomass swelling, disruption of the lignin structure, and consequently leads to an increase in internal surface area and median pore volume for enzyme attack to cellulose [11]. Investigation of compositional and structural changes of lignocellulosic materials after the pretreatment is important to understand the mechanism for improvement of lignocelluloses digestibility and accordingly an optimum pretreatment process could be designed.

Pretreatment of lignocelluloses can be performed by mechanical, chemical, biological processes, or their combinations [12,13]. Biological pretreatment employs microorganisms, mainly white-rot fungi [14,15], brown-rot fungi [16] and bacteria [17], and their enzymatic machineries to break down lignin and alter lignocellulose structures. Biological pretreatment has the potential to be developed and applied industrially, since it has several advantages such as (i) low energy requirement, (ii) low capital investment, (iii) no requirement for chemicals, (iv) mild environmental conditions, (v) substrate specificity, and (vi) simple process and equipment requirements [18]. White-rot fungi, such as *Pleurotus* sp., are intensively investigated for application in biological pretreatment of lignocellulosic materials in the production of biopulps, ruminant feeds, and biofuels [15,19,20]. White-rot fungi produce ligninolytic enzymes, which degrade lignin efficiently [21]. These fungi also degrade holocellulose and decrease the crystallinity of cellulose. In order to increase the performance of the biological pretreatment, fungal pretreatment can be performed in combination with a physical method such as steam explosion [22], or chemical methods such as H_2_O_2_ [23], organosolv [24] or H_2_SO_4_ [25]. Combination of the fungal pretreatment and the physical/chemical pretreatment results in reduced pretreatment duration [22], enhanced delignification [23], and improved enzymatic hydrolysis and ethanol yields [25].

Phosphoric acid pretreatment of lignocellulosic material has been reported to successfully fractionate and enhance digestibility of the lignocelluloses [26]. The present study deals with pretreatment of OPEFB by the fungus *Pleurotus* *floridanus*, phosphoric acid, and a combination of fungal followed by phosphoric acid pretreatments. Phosphoric acid pretreatment was efficient in reducing crystallinity of cellulose and improving biogas yield from OPEFB [27]. Combination of biological pretreatment with phosphoric acid pretreatment was not detected in the literature. Dry weight loss and compositional changes of OPEFB during pretreatment were investigated. Structural changes of OPEFB were analysed using FTIR and SEM, and changes in composition and structure of OPEFB in correlation with digestibility were discussed.

## 2. Results and Discussion

### 2.1. Effect of Pretreatment on Biomass Components

**Figure 1:**
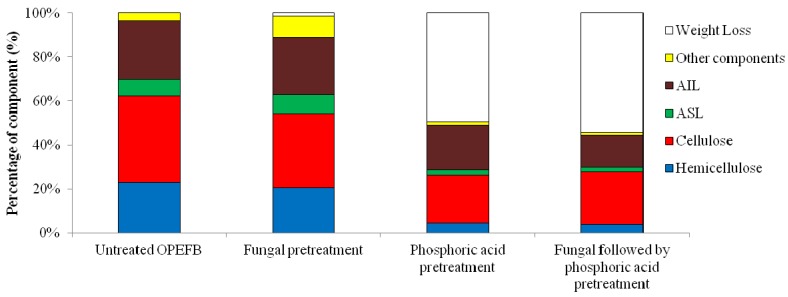
Profile of components of oil palm empty fruit bunches (OPEFB) after pretreatment. ASL: acid soluble lignin, AIL: acid insoluble lignin.

Oil palm empty fruit bunches were pretreated biologically using *P. floridanus* and chemically using phosphoric acid. The composition of OPEFB before and after the pretreatment was analysed and the results were presented in Table 1 and Table 2 and Figure 1. The composition of OPEFB was only slightly changed by the fungal pretreatment (Table 1), but it was significantly altered by phosphoric acid or fungal followed by phosphoric acid pretreatments. Among the components in OPEFB, hemicellulose is the most affected one by the pretreatment, *i.e*., about 18% loss (Table 2 and Figure 1). It is clearly shown that the fungal pretreatment results in the least losses of both total solid (1.31%) and total carbohydrate (7.88%) compared with two other pretreatments.

**Table 1:** Composition of oil palm empty fruit bunches (OPEFB) after pretreatment.

	ASL (%)	AIL (%)	Total Lignin (%)	Cellulose (%)	Hemicellulose (%)	Total Solid Loss (%)
Untreated OPEFB	7.81 ± 0.03	26.56 ± 0.14	34.37 ± 0.17	39.13 ± 2.26	23.04 ± 2.79	0
Fungal pretreatment	8.39 ± 0.40	25.95 ± 0.36	34.34 ± 0.36	34.17 ± 0.40	27.48 ± 9.07	1.31 ± 0.13
Phosphoric acid pretreatment	4.30 ± 0.09	40.13 ± 0.51	44.66 ± 0.18	43.16 ± 0.43	9.07 ± 0.05	54.84 ± 1.37
Fungal followed by phosphoric acid pretreatment	4.53 ± 0.05	32.92 ± 0.60	37.22 ± 0.51	53.81 ± 1.14	9.07 ± 0.14	63.55 ± 0.76

ASL: acid soluble lignin; AIL: acid insoluble lignin; Total Lignin = ASL + AIL.

**Table 2:** Percentage of component loss of OPEFB after pretreatment compared to initial mass.

	ASL (%)	AIL (%)	Total Lignin (%)	Cellulose (%)	Hemicellulose (%)	Total Carbohydrate (%)
Untreated OPEFB	0	0	0	0	0	0
Fungal pretreatment	−0.47 *	0.95	0.48	5.40	2.48	7.88
Phosphoric acid pretreatment	5.51	6.19	11.70	17.22	18.43	35.65
Fungal following phosphoric acid pretreatment	5.87	11.69	17.56	14.83	18.94	33.77

ASL: acid soluble lignin, AIL: acid insoluble lignin, Total Lignin = ASL + AIL; Total Carbohydrate = Cellulose + Hemicellulose; * the negative value of ASL means that the ASL of pretreated sample is higher than the untreated sample, which is probably because of partial conversion of AIL to ASL. Further investigation is required to clarify this fact.

As presented in Table 2, the total solid losses following phosphoric acid pretreatment and fungal followed by phosphoric acid pretreatment are approximately 55% and 64%, respectively, whereas the total carbohydrate losses of both treatments were correspondingly 35% and 33%. Thus, the fungal pretreatment showed more advantageous in maintaining the carbohydrate content than phosphoric acid pretreatment and fungal followed by phosphoric acid pretreatment. Accordingly, using fungal pretreatment, much more lignocellulosic material remains to be utilized, and it has less environmental impacts.

### 2.2. Effects of Pretreatment on the OPEFB Structure

**Figure 2:**
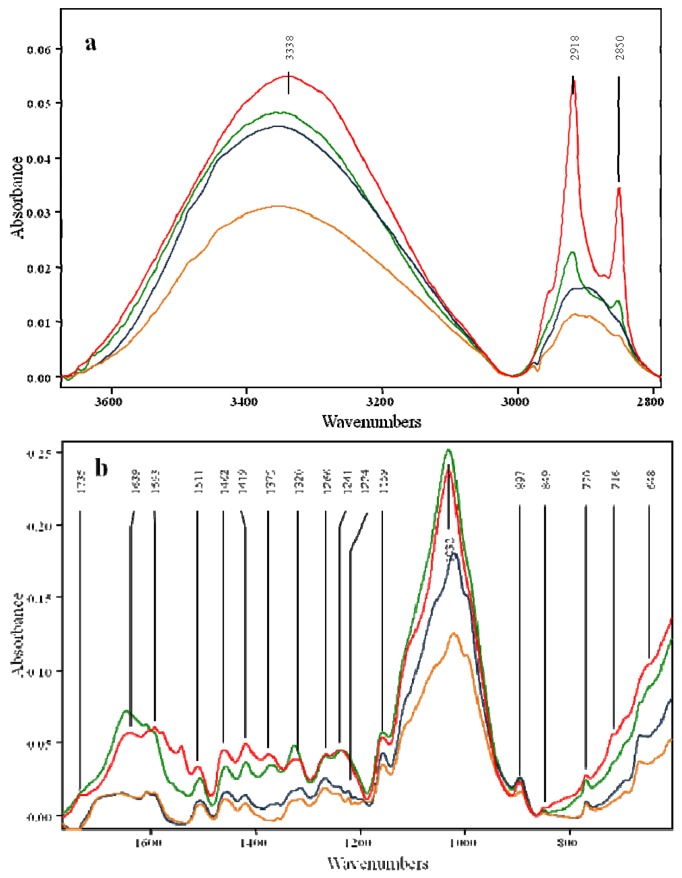
FTIR spectra of oil palm empty fruit bunches (OEPFB) in the wavenumber ranges of 2,800–3,800 cm^−1^ and 600–1,800 cm^−1^. Line assignment: untreated (red line), fungal pretreatment (green line), phosphoric acid pretreatment (light blue line), fungal followed by phosphoric acid pretreatment (light brown line).

The structural changes of OPEFB were analysed based on FTIR spectra of the untreated and pretreated materials. The results are shown in Figure 2. Band assignments and band shifts according to the literature are listed in Table 3. Fourteen bands were conserved in all of the samples in the range of 600–1,800 cm^−1^ and 2,800–3,700 cm^−1^. Bands at wavenumbers 648, 2,918, and 2,985 cm^−1^ with high intensity were only found in untreated and fungal pretreated OPEFB. Bands that only appeared in samples pretreated with phosphoric acid and fungal followed by phosphoric acid were 1,224, 998 and 666 cm^−1^.

A strong and broad absorption was observed at a wavenumber of around 3,300 cm^−1^. This wavenumber was assigned to hydrogen bonded (O-H) stretching absorption. O-H stretching region at a wavenumber of 3,000–3,600 cm^−1^ of OPEFB spectra was more identical to the O-H stretching region from cellulose I than cellulose II. The valence vibration of hydrogen-bonding of OH groups of cellulose I is the sum of three different hydrogen-bonds: intramolecular hydrogen bond of 2-OH···O-6, intramolecular hydrogen bond of 3-OH···O-5, intermolecular hydrogen bond of 6-OH···O-3 [28]. Relative band-height at this region decreased as cellulose content decreased.

The highest cellulose loss was observed in fungal followed by phosphoric acid pretreatment as indicated with the lowest intensity on O-H stretching absorption. A strong intensity band at wavenumbers 2,985 and 2,918 cm^−1^ was found in untreated and fungal pretreated OPEFB.

**Table 3:** Assignments of IR band maxima to various components of oil palm empty fruit bunches according to literature.

Untreated OPEFB	Fungal pretreatment	Phosphoric acid pretreatment	Fungal followed by phosphoric acid pretreatment	Assignments	Source	Ref.
Wavenumber (cm^−1^)						
648	666	666	667	C-O out-of-plane bending mode	Cellulose	[29]
716	-	-	-	Rocking vibration CH_2_ in Cellulose I_β_	Cellulose	[29]
770	770	769	769	CH_2_ vibration in Cellulose I_α_	Cellulose	[29]
849	851	850	851	C-H out of plane deformation in position 2,5,6	G-Lignin	[30]
897	896	895	895	Anomere C-groups C(1)-H deformation, ring valence vibration	Polysaccharides	[30,31]
-	-	998	997	C-O valence vibration		[29]
1,032	1,033	1,022	1,022	Aromatic C-H in plane deformation, G > S; plus C-O deformation in primary alcohols; plus C=O stretch (unconj.)	Lignin	[29]
1,159	1,159	1,158	1,158	C-O-C assimetric valence vibration	Polysaccharides	[30]
-	-	1,224	1,223	C-C plus C-O plus C=O strech; G condensed > G etherified	Polysaccharides	[30,31]
1,241	1,237	1,243	1,245	C=O stretch, OH i.p. bending		[32]
1,266	1,267	1,267	1,267	G-ring plus C=O strectch	G-Lignin	[33]
1,321	1,326	1,315	1,315	O-H blending of alcohol groups	Carbohydrate	[30]
1,375	1,371	1,370	1,372	C-H deformation vibration	Cellulose	[31]
1,418	1,418	1,420	1,419	Aromatic skeletal vibrations with C-H in plane deformation CH2 scissoring	Lignin	[34]
1,462	1,457	1,455	1,459	C-H in pyran ring symmetric scissoring; OH plane deformation vibration	Cellulose	[31]
1,511	1,507	1,506	1,506	Aromatic skeletal vibrations;G > S	Lignin	[34]
1,593	1,609	1,608	1,607	Aromatic skeletal vibrations plus C=O stretch; S > G; G condensed > G etherified	Lignin	[34]
1,640	1,646	1,654	1,663	C O stretch in conjugated p-substituted aryl ketones	Lignin	[34]
1,735	1,735	1,735	1,735	CO stretch unconjugated (xylan)	Polysaccharides	[34]
2,850	2,850	2,850	2,850	Asymetric CH2 valence vibration		[29]
2,918	2,918	2,918	2,918	Symmetric CH2 valence vibration		[29]
3,338	3,345	3,346	3,351	Hydrogen bonded O-H valence vibration; O(3)H...O(3) intermolecular in cellulose	Cellulose	[29]

However, it shows a shoulder peak at these wavenumbers after phosphoric acid pretreatment and fungal followed by phosphoric acid pretreatment. The band in the region of 2,985 cm^−1^ was assigned to the asymmetric stretch vibration of CH_2_ from the CH_2_-OH group in cellulose, and the band in the region of 2,918 cm^−1^ was derived from the symmetric vibration of the CH_2_ group. The strong intensity at these two wavenumbers are similar to IR spectra from hardwood and hardwood lignin [35], which indicates that lignin structure in OPEFB is similar to hardwood lignin.

IR spectra at the range of wavenumbers 1,150 and 1,750 cm^−1^ clearly showed two distinct spectra groups (Figure 2). The band at a wavenumber of around 1,735 cm^−1^ was assigned to an unconjugated carbonyl originated from the uronic acid of the xylans in hemicellulose [34]. In this peak, there may exist linkages between lignin and carbohydrate [36]. IR intensities at this wavenumber diminished after fungal pretreatment. Interestingly, it showed shoulder peaks after phosphoric acid pretreatment and fungal followed by phosphoric acid pretreatment. These peaks at wavenumber 1,735 cm^−1^ confirmed slight changes in hemicellulose content after fungal pretreatment and a high loss of hemicellulose after phosphoric acid pretreatments and fungal followed by phosphoric acid pretreatment (Figure 1).

Structural changes in lignin and loss of aromatic units were shown by the intensities in the changes in the 1,646, 1,593 and 1,506 cm^−1^ bands. Fungal pretreatment increased the intensity of the 1,646 cm^−1^ band and decreased the intensity of the bands at 1,593 and 1,506 cm^−1^. These changes suggest a split between the benzylic α- and β-carbon atoms by fungal pretreatment [34]. Both phosphoric acid pretreatment and fungal followed by phosphoric acid pretreatment showed similar intensities for the bands at 1,646, 1,607, 1,593, and 1,506 cm^−1^. These spectra explained the fact shown in Table 1 and Table 2 that pretreated OPEFB by phosphoric acid pretreatment and fungal followed by phosphoric acid pretreatment had a similar loss and the percentage of ASL.

IR intensity decreased at wavenumbers 1,462 and 1,418 cm^−1^, but increased at wavenumber 1,321 cm^−1^ after fungal pretreatment. IR intensities of these bands were reduced after phosphoric acid pretreatment and fungal followed by phosphoric acid pretreatment. Different intensities were also found in the bands near wavenumbers 1,267 and 1,236 cm^−1^. The intensities at these bands did not change after fungal pretreatment, but reduced after phosphoric acid pretreatment. A band at 1,267 cm^−1^ was assigned to the guaiacyl of lignin. A band at 1,235 cm^−1^ was attributed to a combination of a deformation of syringyl and cellulose. The decrease in intensity at wavenumber 1,235 cm^−1^ was greater than that at wavenumber 1,267 cm^−1^ after phosphoric acid pretreatment. This suggests that syringyl was more solubilized by phosphoric acid than guaiacyl lignin.

The band at wavenumber 1,375 cm^−1^ was assigned to C-H deformations in cellulose and hemicellulose. The intensity of this band was slightly decreased after fungal pretreatment and it showed a slight loss of cellulose and hemicellulose content. A higher decrease in intensity was found after phosphoric acid pretreatment, which could be related to the high loss of hemicellulose content. Decreasing intensities were also found in the band at wavenumber 1,159 cm^−1^, which was assigned to C-O-O- > C-O-C asymmetric vibration of cellulose and hemicellulose. All the pretreated OPEFB samples showed lower intensities than the untreated OPEFB. Changes in intensity were also found in the band at around wavenumber 1,032 cm^−1^ that was assigned to the C-O stretch in cellulose and hemicellulose. Intensity of this band was slightly increased after fungal pretreatment. On the other hand, it shifted to 1,021 cm^−1^ and decreased in intensity after phosphoric acid pretreatment. The shifting and decreasing at this band might be attributed to decreased hemicellulose content after phosphoric acid pretreatment.

**Figure 3:**
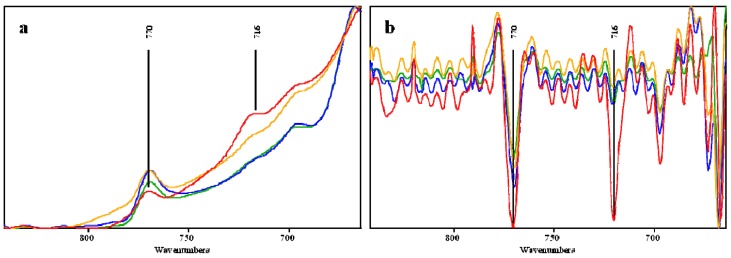
FTIR spectra (**a**) and second derivative spectra (**b**) at wavenumber 770 cm^−1^ (CH_2_ vibration in cellulose *I_α_*) and 716 cm^−1^ (CH_2_ vibration in cellulose *I_β_*). Lines assignment were: untreated (red line), fungal pretreatment (green line), phosphoric acid pretreatment (light blue line), fungal followed phosphoric acid pretreatment (light brown line).

The peak at a wavenumber around 895 cm^−1^ was assigned to C-H-O stretching of the β-(1-4)-glycosidic linkage. Intensities of this peak were increased after fungal pretreatment and phosphoric acid pretreatment, but decreased by fungal followed by phosphoric acid pretreatment. Figure 3 shows peaks at wavenumbers of around 750 cm^−1^ and 716 cm^−1^ that were assigned to rocking vibration CH_2_ in cellulose I_α_ and cellulose I_β_, respectively. Crystalline cellulose I is composed of two allomorphs, cellulose *I_α_* (triclinic) and cellulose *I_β_* (monoclinic) [37]. Peaks at 769 cm^−1^ were clearly observed in all spectra. A clear peak at wavenumber 716 cm^−1^ was only found in untreated OPEFB spectra which then became a shoulder peak after pretreatments. Second derivative spectra revealed that peaks at a wavenumber around 769 cm^−1^ for cellulose I_α_ showed constant intensities after pretreatment. However, peaks at a wavenumber of 716 cm^−1^ for cellulose I_β_ were decreased significantly after pretreatment (Figure 3).

Different methods have been proposed to characterize and quantify the crystallinity of cellulose using the ratio of the intensities of certain bands at the IR spectra, *i.e*., 2,900, 1,429, 1,372, 894 and 670 cm^−1^ [34,38,39,40]. The IR A1418/A895 known as Lateral Order Index (LOI) is the ratio between absorbance at wavenumber 1,418 and 895 cm^−1^ [40,41]. LOI value of untreated, fungal pretreated, phosphoric acid pretreated, and fungal followed by phosphoric acid pretreated OPEFB are 2.78, 1.42, 0.67, and 0.60, respectively. Untreated OPEFB has the highest value and the greatest decrease was achieved by phosphoric acid pretreatment. There is no significant difference between the LOI values of phosphoric acid and fungal pretreatment followed by phosphoric acid pretreatment. The LOI showed a linear correlation with the hemicellulose content. The correlation of LOI and hemicelluloses was probably due to the fact that the band at 894 cm^−1^ was assigned to the anomeric carbon group frequency in hemicellulose and cellulose [42].

### 2.3. Effect of Pretreatments on OPEFB Morphology

**Figure 4:**
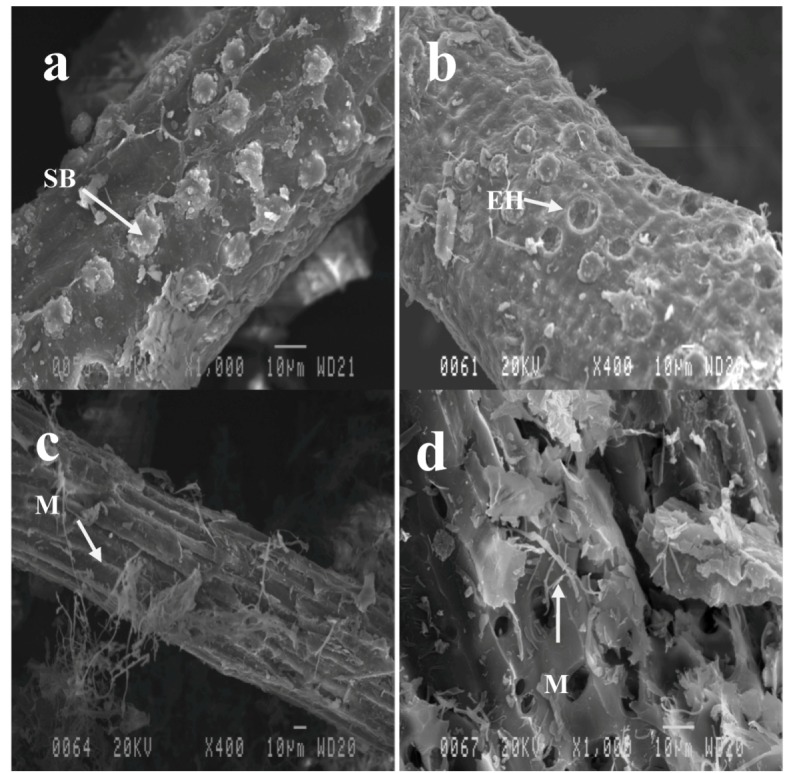
Fibre surface of untreated Oil palm empty fruit bunches (OPEFB). (**a**) Untreated OPEFB, (**b**) fungal pretreated OPEFB, (**c**) fungal pretreated OPEFB strand covered by fungal mycelium, (**d**) cross section of fungal pretreated OPEFB. SB = silica body, EH = empty hole, M = mycelium.

Photomicrographs of untreated OPEFB and fungal-pretreated OPEFB are presented in Figure 4. The strand surface of untreated OPEFB has round-shaped spiky silica-bodies. The silica bodies were found in great number and attached relative uniformly around the fibre surface. Fungal pretreated OPEFB shows that some of silica bodies were removed from the strand surface and left empty holes at the bottom of silica-bodies creatures (Figure 4b). The surfaces of fungal pretreated OPEFB are rugged and partially broken faced. Mycelium growth was found in fungal pretreated OPEFB (Figure 4c,d). Mycelium grows outside and penetrates inside the OPEFB strand.

**Figure 5:**
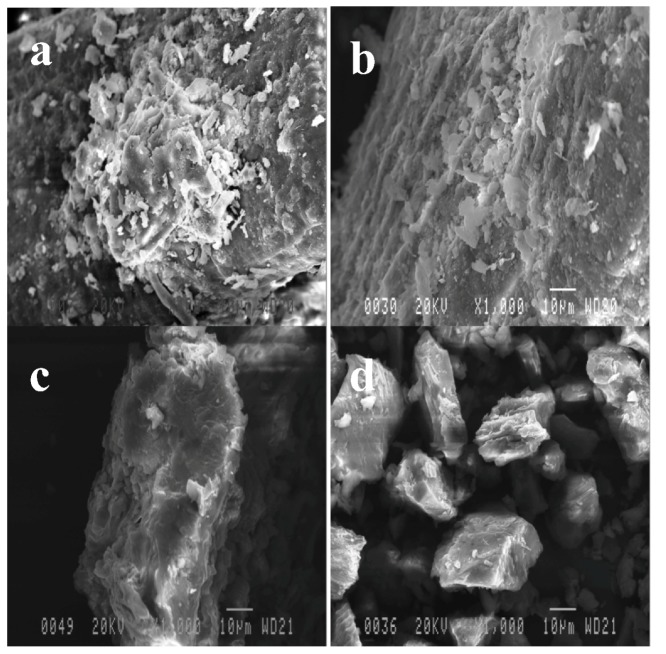
Morphological changes of OPEFB surface before and after pretreatment. All samples were size-reduced using ball milling before hydrolysis and fermentation. (**a**) Untreated OPEFB, (**b**) fungal pretreated OPEFB, (**c**) phosphoric acid pretreated OPEFB, (**d**) fungal followed by phosphoric acid pretreated OPEFB.

Prior to hydrolysis, pretreated OPEFB was ball-milled to reduce the particle size in order to increase the surface area for enzymes to attack. Figure 5 presents photomicrographs of untreated, fungal pretreated, phosphoric acid pretreated, and fungal followed by phosphoric acid pretreated OPEFB after being ball-milled. The particle size of pretreated OPEFB varied. Untreated and fungal-pretreated OPEFB showed larger particle size compared to OPEFB pretreated by phosphoric acid and fungal followed by phosphoric acid pretreatment (Figure 5a,b). Some silica bodies were partially removed in the untreated OPEFB, but the removal was higher in the fungal pretreated OPEFB. The silica bodies are hard, but can be removed mechanically, e.g., hammering or milling [2]. It seems that silica bodies were easier to remove by ball mill in the fungal pretreated OPEFB than in the untreated OPEFB. Phosphoric acid and fungal followed by phosphoric acid pretreated samples shows small size and non-uniform particles (Figure 5c,d). Particle sizes of the phosphoric acid pretreated samples were bigger than that of fungal followed by phosphoric acid pretreated samples. Strands of OPEFB are completely broken after phosphoric acid pretreatment and fungal followed by phosphoric acid pretreatment. The photomicrographs revealed that strands of OPEFB pretreated by phosphoric acid and fungal followed by phosphoric acid pretreatment were weaker and easier to grind than OPEFB strands pretreated by the other methods.

### 2.4. Cellulose Digestibility of Untreated and Pretreated OPEFB

**Figure 6:**
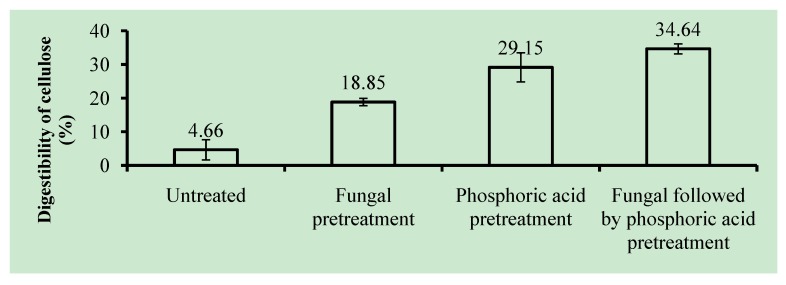
Digestibility of cellulose (%) of oil palm empty fruit bunches (OPEFB) in the enzymatic hydrolysis process (based on initial cellulose content after pretreatments). Error bars are standard deviation.

Figure 6 shows the digestibility of untreated and pretreated OPEFB after 72 h enzymatic hydrolysis. The digestibility was calculated based on initial cellulose content prior to hydrolysis. It is shown that untreated OPEFB had very low digestibility (4.66%), which could be because of its high lignin and high hemicellulose contents, as well as high crystallinity of cellulose.

The digestibility of OPEFB after fungal, phosphoric acid, and fungal followed by phosphoric acid pretreatment was 18.85%, 29.15%, and 34.64%, respectively [calculated following Equation (1) in Section 3.5 with the initial cellulose value taken before pretreatments]. These values correspond to an increase in digestibility by 400%, 630%, and 740% compared to that of the untreated OPEFB. If digestibility is calculated based on initial cellulose content after pretreatment, the corresponding values for the pretreated OPEFB would be 19.10%, 64.56%, and 69.12%. The difference in digestibility values can be explained by the profile of the composition of OPEFB after pretreatment that showed significant loss of cellulose content up to 63.55% (Table 2). Furthermore, those values are comparable with the digestibility of OPEFB after Ammonia Fibre Expansion (AFEX) pretreatment (58%) [43], alkaline pretreatment (69.69%) [4], superheated steam pretreatment (66.33%) [44] and sodium hydroxide—sodium hypochlorite pretreatment (60%) [45].

As mentioned previously, LOI of untreated, fungal-treated, phosphoric acid-treated, and fungal-phosphoric acid-treated OPEFB were 2.78, 1.42, 0.67, and 0.60, respectively. This implies that the digestibility of OPEFB after pretreatment has an inverse correlation with LOI. Digestibility is enhanced as crystallinity of the cellulose is reduced as shown by the lower LOI value. The IR spectra of the fungal-pretreated materials indicate that the fungus might attack the linkages between lignin and carbohydrate that exist in hemicellulose. These linkages were observed in the IR spectra at a wavenumber of 1,735 cm^−1^ [31]. The reduction in the intensity of this peak as well as a reduction in hemicellulose and linkages might have contributed to the improved digestibility of the OPEFB. Fungal pretreatment of the OPEFB reduced cellulose *I_β_* as shown in IR spectra, to a proportion of *I_α_* greater than *I_β_*. Cellulose *I_α_* is meta-stable and more reactive than *I_β_* [37]. It might make the OPEFB more reactive and easier to be hydrolysed. 

One of the advantages of phosphoric acid pretreatment is the reduced crystallinity of the cellulose [46]. Crystallinity of the OPEFB decreased after both phosphoric acid pretreatments and fungal followed by phosphoric acid pretreatment, which might result in improved digestibility. Phosphoric acid pretreatment also reduced cellulose *I_β_* as shown in IR spectra. Fungal followed by phosphoric acid pretreatment gave higher digestibility than phosphoric acid pretreatment. A higher digestibility improvement of fungal followed by phosphoric acid pretreatment than the phosphoric acid pretreatment was confirmed by a reduction in both crystallinity and cellulose *I_β_*. Furthermore, OPEFB pretreated by phosphoric acid and fungal followed by phosphoric acid methods showed relatively high lignin proportion up to 44.66% (Table 1). This finding stresses the fact that lignin seems not the only recalcitrant factor of OPEFB. Available surface area and accessibility to cellulose of OPEFB after pretreatment contribute to improved digestibility of lignocellulosic materials [47].

## 3. Experimental

### 3.1. Oil Palm Empty Fruit Bunch

OPEFB was collected from an oil palm mill in North Sumatera, Indonesia. Fresh OPEFB were chopped and air-dried until the moisture content was less than 10%. Dried OPEFBs were ground to pass through a 10-mm screen and stored in a container at room temperature prior to pretreatment. It was analysed for its lignin, cellulose, and hemicelluloses contents.

### 3.2. Microorganism

*P.* *floridanus* strain LIPIMC996 (Laboratory of Microbial Systematic and LIPI Microbial Collection, Lembaga Ilmu Pengetahuan, Cibinong, Indonesia) was used in this work. The fungus was maintained on lignocellulosic medium at room temperature prior to use as inoculums. Small pieces of active mycelia were cut from fungal culture on lignocellulosic medium and were inoculated aseptically into potato dextrose agar (PDA, Difco Laboratories, Detroit, MI, USA) plates and incubated at room temperature. The culture of *P. floridanus* was re-cultured for one to two weeks and used as inoculums. 

### 3.3. Biological Pretreatment

For the biological pretreatment, a mixture of 200 g of OPEFB and 120 mL of medium (containing 7 g/L KH_2_PO_4_, 1.5 g/L MgSO_4_.7H_2_O, 1 g/L CaCl_2_·H_2_O, 0.3 g/L MnSO_4_.H_2_O, and 0.3 g/L CuSO_4_·H_2_O) was autoclaved at 121 °C for 1 h. The medium was added to adjust the water content of OPEFB to 70%. The mixture of OPEFB and medium was inoculated with a half agar plate culture of two week old inoculums and incubated at 31 °C for 28 days. At the end of incubation, OPEFB was harvested and frozen to terminate fungal growth. OPEFB samples were freeze-dried (Freezone 7670530, Labconco, Kansas City, MO, USA) at −52 °C for 6 h and then ball milled (Retsch^®^ MM400, Retsch GmbH, Haan, Germany) at a frequency of 29.6 s^−1^ for 4 min prior to enzymatic hydrolysis. Dry weight loss was determined as the difference of the oven dry weight (ODW) of OPEFB at the beginning and at the end of the pretreatment process. All experiments were carried out in duplicate.

### 3.4. Phosphoric Acid Pretreatment

The pretreatment using phosphoric acid was carried out for untreated and fungal-treated OPEFB in accordance with the method described in references with slight modification [26,27]. Prior phosphoric acid pretreatment, the untreated and fungal-treated OPEFB were ball-milled (Retsch^®^ MM400) at a frequency of 29.6 s^−1^ for 4 min. One gram of OPEFB was mixed with 8 mL of phosphoric acid (85.7%) in a 50 mL-centrifuge tube and stirred using a glass rod until it was homogenised. The mixture was then incubated in a shaker bath (Grant OLS200, Grant Instruments Ltd., Cambridgeshire, UK) at 90 rpm and 50 °C for 5 h. The reaction was terminated with the addition of 20 mL acetone and centrifuged at 3,400 rpm for 15 min. Solid pellets were washed by adding 40 mL acetone and centrifuged three times to remove all the residual acid. Acid free residual pellets were then washed three times using 40 mL distilled water and centrifuged until a clear supernatant with pH 7 was obtained. Treated samples were frozen before use in the hydrolysis process. The same procedure was applied for fungal treated OPEFB.

### 3.5. Enzymatic Hydrolysis

Enzymatic hydrolysis of untreated and treated OPEFB was performed based on the National Renewable Energy Laboratory (NREL) method with slight modification [48]. Commercial Cellic^®^ CTec2 (148 FPU/mL, Novozymes Co., Bagsvaerd, Denmark) were used in this experiment. The treated and untreated OPEFB were hydrolyzed in a 50 mL-Erlenmayer flask with a working volume of 10 mL at pH 4.8 (citric acid buffer 0.05 M), at 50 °C and agitated at 150 rpm for 72 h. Enzyme loadings were 30, 60, and 90 FPU/g cellulose. The digestibility (%) was calculated by dividing the glucose produced by the initial cellulose used based on the following equation:

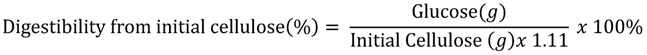
(1)
where glucose (g) is the amount of glucose in the liquid after hydrolysis and initial cellulose (g) is the amount of cellulose in the substrates, either before or after pretreatments. All experiments were conducted in duplicate and the error was presented as standard deviation.

### 3.6. Analytical Methods

The cellulose, hemicellulose, and lignin of the OPEFB were determined according to the NREL methods [49]. The materials were hydrolysed by two-step hydrolysis using 72% H_2_SO_4_ at 30 °C for 60 min for step one, continued with step two using 4% H_2_SO_4_ at 121 °C for 60 min in autoclave. Monomeric sugars contained in the liquid were determined by liquid chromatography. A HPLC system was equipped with an autosampler (Waters^TM^ 717, Milford, MA, USA), UV detector (Waters^TM^ 485), and an ELS detector (Waters^TM^ 2424). Sugars were analysed using a lead-based column (Aminex HPX-87P, Bio-Rad, Hercules, CA, USA), pure water as mobile phase at a flow rate of 0.6 mL min^−1^ under isothermal condition at 85 °C. Acid insoluble lignin (AIL) was determined gravimetrically as a residual solid after hydrolysis and corrected with ash content. Acid Soluble lignin (ASL) was determined using a spectrophotometer at a wavelength of 240 nm and 25 L/(g.cm) as ε value. Total solids were determined by drying samples at 105 °C overnight until a constant weight was obtained. The amount of ash was determined using a furnace overnight at 575 °C [50].

A Fourier transform infrared (FTIR) spectrometer (Impact 410 iS10, Nicolet Instrument Corp., Madison, WI, USA) was used for determining changes in the structure of the OPEFB after the pretreatments. Each spectrum was obtained with an average of 32 scans and resolution of 4 cm^−1^ from 600–4,000 cm^−1^ [46]. The spectrum data was controlled by Nicolet OMNIC 4.1 (Nicolet Instrument Corp.) software and analysed by eFTIR^®^ (EssentialFTIR, Operant LLC, Sarasota, FL, USA). The baseline of all spectra was corrected so that the minimum point at wavenumber 3,674, 3,008, 1,775 and 820 cm^−1^ was set to zero. The four wavenumbers were chosen because the correction of the baseline made the most of minimum peaks of fingerprint band close to zero. All spectra were normalized by the highest absorbances.

To evaluate the changes in the surface of OPEFB before and after pretreatment, samples were visualized using scanning electron micrograph (SEM), model Jeol JSM-820 (Jeol Ltd., Akishima, Japan). Vacuum dried of untreated and pretreated OPEFB was powdered over a specimen stub (10 mm in diameter) covered with a carbon conductive tab (doubled coated, 12 mm in diameter, PELCO Tabs). Excess sample was removed by turning the specimen stub upside down and gently shaking the stub. Before samples were mounted into the SEM instrument, the surfaces of the samples were coated with a thin (approximately 20 nm thick) of gold in an ion sputter, model JFC-1100E (Jeol Ltd.). SEM images were recorded at 1,000× magnification using an acceleration voltage of 20 kV.

## 4. Conclusions

Composition of OPEFB was changed by fungal, phosphoric acid and fungal followed by phosphoric acid pretreatments. Fungal pretreatment resulted in slight reduction of all components of OPEFB. On the other hand, phosphoric acid and fungal followed by phosphoric acid pretreatment reduced significant amounts of hemicellulose and cellulose. Structural and morphological changes of OPEFB after pretreatments were confirmed by FTIR and SEM analysis. SEM analysis showed that OPEFB’s fiber structures were completely damaged after phosphoric acid and fungal followed by phosphoric acid pretreatment. Important structural changes observed by FTIR analysis are reduction in hydrogen bonded (O-H) stretching absorptions, unconjugated carbonyl absorptions, absorption of peaks assigned to cellulose and hemicellulose, and decreases in the peaks of cellulose I_β_. Hemicellulose loss, reduced cellulose I_β_, and reduction of particle size might be responsible for increasing digestibility of OPEFB.
